# Longitudinal Associations Between Disaster Damage and Falls/Fear of Falling in Older Adults: 9-Year Follow-Up of Survivors of the 2011 Great East Japan Earthquake and Tsunami

**DOI:** 10.1093/geroni/igad020

**Published:** 2023-03-01

**Authors:** Yuhang Wang, Chenggang Zhang, Hiroyuki Hikichi, Ichiro Kawachi, Xiaoyu Li

**Affiliations:** Department of Sociology, Tsinghua University, Beijing, China; Department of Sociology, Tsinghua University, Beijing, China; School of Medicine, Kitasato University, Sagamihara, Kanagawa, Japan; Department of Sociology, Tsinghua University, Beijing, China; Department of Social and Behavioral Sciences, Harvard T.H. Chan School of Public Health, Boston, Massachusetts, USA; Department of Sociology, Tsinghua University, Beijing, China

**Keywords:** Cumulative disadvantage, IADL, Postdisaster material hardship, Social capital

## Abstract

**Background and Objectives:**

Fear of falling and falls are common in older adults. However, their associations with natural disaster exposures remain poorly understood. This study aims to examine longitudinal associations between disaster damage with fear of falling/falls among older disaster survivors.

**Research Design and Methods:**

In this natural experiment study, the baseline survey (4,957 valid responses) took place 7 months before the 2011 Great East Japan Earthquake and Tsunami, and 3 follow-ups were conducted in 2013, 2016, and 2020. Exposures were different types of disaster damage and community social capital. Outcomes were fear of falling and falls (including incident and recurrent falls). We used lagged outcomes in logistic models adjusting for covariates and further examined instrumental activities of daily living (IADLs) as a mediator.

**Results:**

The baseline sample had a mean (standard deviation) age of 74.8 (7.1) years; 56.4% were female. Financial hardship was associated with fear of falling (odds ratio (OR), 1.75; 95% confidence interval (CI) [1.33, 2.28]) and falls (OR, 1.29; 95% CI [1.05, 1.58]), especially recurrent falls (OR, 3.53; 95% CI [1.90, 6.57]). Relocation was inversely linked with fear of falling (OR, 0.57; 95% CI [0.34, 0.94]). Social cohesion was protectively associated with fear of falling (OR, 0.82; 95% CI [0.71, 0.95]) and falls (OR, 0.88; 95% CI [0.78, 0.98]) whereas social participation increased the risk of these issues. IADL partially mediated observed associations between disaster damage and fear of falling/falls.

**Discussion and Implications:**

Experiences of material damage rather than psychological trauma were associated with falls and fear of falling, and the increased risk of recurrent falls revealed a process of cumulative disadvantage. Findings could inform targeted strategies for protecting older disaster survivors.

**Translational significance:** Fear of falling and falls are common and major concerns in older adults. However, little is known on their associations with natural disaster exposures. In this prospective cohort study, we found that experiences of material damage were associated with fear of falling and falls, whereas the increased risk of recurrent falls showed a process of cumulative disadvantage. Social cohesion was protectively associated with fear of falling and falls. The findings could inform targeted disaster recovery efforts by drawing attention to material support, social attachment, and high-risk populations to mitigate lingering fall-related problems among older disaster survivors.

## Background and Objectives

Falls and fear of falling in older adults are major and under-recognized public health concerns in this era of aging (Romli et al., 2017; World Health Organization, 2021). Approximately 684,000 people die from falls each year globally (James et al., 2020), making falls the second leading cause of unintentional injury-related fatalities (Roth et al., 2018). Fear of falling is often entangled with falls and also easily leads to severe physical and psychosocial consequences (Friedman et al., 2002; Liu et al., 2021; Sawa et al., 2020). Previous studies have linked fear of falling and falls to personal characteristics (Chen et al., 2012; Deandrea et al., 2010; Montero-Odasso et al., 2021; Scheffer et al., 2008). Musculoskeletal disease, eye disease, and functional disability could give rise to fear of falling and falls (Deandrea et al., 2010; Vlaeyen et al., 2017). The importance of dwelling environments, including house story, lighting quality, and leveling of sidewalks, has been also highlighted (Canever et al., 2021; Peek-Asa et al., 2003). Moreover, researchers have documented the role of social capital (defined as network features that facilitate coordination for mutual benefit and provide resources that are accessed by memberships; Kawachi et al., 2000; Putnam, 1995) in preventing fear of falling and falls (Howland et al., 1998; Okoye et al., 2021).

From a gerontological perspective, it is notable that natural disasters erupt frequently in current times (Curran, 2013) and increasingly affect older populations (Li et al., 2019). Older adults are vulnerable to disasters (Sands et al., 2022; Tome et al., 2022). When disasters strike, it is difficult for some older adults to pay attention to and comprehend warning messages due to their sensory impairments in visual and hearing capacities (Mayhorn, 2005). With age-related mobility limitations and decreased social resources, some of them may also be less able to take self-protective measures (Hattori et al., 2021). A considerable proportion of older adults have chronic conditions that, if left untreated or without a prescription after a disaster, could deteriorate and become life threatening (Chang et al., 2022). Additionally, numerous disaster-related stressors have been proven to be linked with disorders of metabolic functions, organ aging, parasecretion, as well as the disruption of healthy lifestyle behaviors (Fukuda et al., 1999; Kivimaki et al., 2022; Razzoli et al., 2018). Hence, research on the impact of disasters specific to older adults is of rising importance (Hikichi et al., 2020; Sands et al., 2022). Along this line of research, previous studies have demonstrated that disaster exposure is strongly associated with postdisaster health problems such as cardiometabolic risks, arthritis, diabetes, dementia, and depression among older adults (Ikeda et al., 2020; Lee et al., 2016; Sharma et al., 2008; Shiba et al., 2019).

However, there is a critical gap in the literature documenting the associations between disaster damage with fear of falling/falls, which are major health concerns among older adults. According to Lawton’s person–environment fit theory, the behaviors and health of older adults are a consequence of the interaction between their capabilities and the pressure of the environment (Lawton et al., 1973; Schlomann et al., 2020). Fear of falling/falls may come from the incongruence between an older adult’s functional ability and features of the environment (Iwarsson et al., 2009), both of which are potentially associated with disasters (Tsuboya et al., 2017). On the one hand, physical and social environment barriers might markedly increase as a result of a disaster. In relation to physical environment, disasters often make people’s residence damaged (e.g., soaked floor and uneven doorways) and alter their living conditions (Li et al., 2018). Besides, survivors’ social environment might be less accessible due to the relocation or loss of intimate friends (Gero et al., 2020). On the other hand, older people’s physical functional abilities might deteriorate under the impact of disasters (e.g., restricted mobility and disrupted health care; Liao et al., 2011; Thiamwong et al., 2017). Mental disorders after a disaster (e.g., cognitive decline, anxiety, and depression) might also increase the risk of functional disability (Bernstein et al., 2022; Ojagbemi et al., 2022). In summary, postdisaster environmental barriers and survivors’ diminished capacities could lead to person–environment incongruence and thus increase the risk of fear of falling/falls (Iwarsson et al., 2009; Wahl et al., 2009). From a clinical perspective, functional disability as stated could be a major risk factor for postdisaster person–environment incongruence (Iwarsson, 2005) and may mediate the relationships between disaster damage and fear of falling/falls. It is also worth noting that moving from a multistory home into a single-story trailer (temporary residence after disasters; Shiba et al., 2020) or simply staying at home more might possibly reduce environmental uncertainties (Plaut et al., 2021). A logical consequence is that the probability of falls could decrease in the circumstance. Hence, the net effect of natural disaster on fear of falling and falls would be the balance between risk and protective aspects.

This study sought to investigate the disaster determinants of fear of falling and falls with longitudinal data from older survivors of the 2011 Great East Japan Earthquake and Tsunami. The study took advantage of a unique natural experiment that collected individuals’ health information 7 months before the disaster and re-contacted the survivors for three follow-ups up to 9 years after the disaster, which gave us the opportunity to prospectively study the predictors of postdisaster fear of falling and falls. The primary aim of this study was to determine the unique impact of various types of disaster damage (including home destruction, financial hardship, health care disruption, home relocation, and loss of close relatives or friends) on fear of falling and falls among older disaster survivors. We hypothesized that each type of disaster damage would predict a higher risk of fear of falling and falls among older survivors. Functional disability could be a potential mediator between these relationships. A secondary aim was to examine the associations between community social capital (including social cohesion, social participation, and reciprocity) and fear of falling and falls. We hypothesized that the availability of each form of social capital would be associated with lower odds of fear of falling and falls over 9 years of follow-up.

## Research Design and Methods

### Study Design and Participants

This study used data from the Japan Gerontological Evaluation Study (JAGES), a national population-based cohort that was established in 2010 to prospectively investigate health risk factors among community-dwelling Japanese older citizens. Our study area, Iwanuma City in Miyagi Prefecture, was one of the field sites of the JAGES cohort. In August 2010, the research team mailed questionnaires to every resident aged 65 years or older, using the official residential register of Iwanuma. A total of 5,058 participants in Iwanuma responded to the survey (response rate = 59.0%) and 4,957 responses were valid. Notably, 7 months after the baseline survey, the 2011 Great East Japan Earthquake and Tsunami directly affected Iwanuma City, located 80 km to the west of the earthquake epicenter. The disaster killed 187 residents (out of a total population of 44,187), damaged 5,428 buildings, and inundated 48% of the land area in Iwanuma (Ishigaki et al., 2013). Given the extensive impact of the earthquake and tsunami, participants in Iwanuma were all disaster survivors who experienced different types of disaster damage. After the disaster, our research team located the addresses of surviving members of the baseline cohort and conducted three follow-up surveys in Iwanuma (October 2013, November 2016, and January 2020). The number of participants in the follow-up survey was 3,567 in 2013; 3,255 in 2016; and 2,545 in 2020, separately. The survey inquired about participants’ experiences during and after the disaster. We included participants observed for at least one follow-up into our analytic sample, keeping as much information as possible.

The study protocol was reviewed and approved by the human subjects committee of the Harvard T.H. Chan School of Public Health, as well as the human subjects committees of Tohoku University and Chiba University. Participants provided written informed consent.

### Measures

#### Outcomes

Two primary outcomes were fear of falling and falls. Fear of falling was evaluated using the question “Are you very worried about falls?” Fall experiences were measured using the question “Have you had any falls over the past year?” These questions were surveyed in all follow-ups. We also investigated two subtypes of fall experiences, incident fall and recurrent falls, for further analyses. Incident fall indicated there was no previous fall experience before this fall accident during the study period. Recurrent falls were defined as at least two fall experiences during the study period (Kabeshova et al., 2014). The fall-related outcomes were binary. All the outcomes were lagged to establish temporality. Because disaster damage was random and exogenous, baseline falls could not cause disaster damage and were thus not treated as confounders.

#### Exposures

The main exposures were experiences of disaster damage and community social capital. Disaster damage variables were measured by a series of questions asking the respondents about their disaster-related experiences in the 2013 survey. Home destruction was measured by asking whether respondents’ residences were damaged by the disaster. Financial hardship was assessed by asking respondents if their financial conditions changed after the disaster. Those who reported “worse” or “much worse” financial conditions were defined as having hardship. Health care disruption was defined as “unable to receive a medical examination because of the disaster.” Home relocation assessed whether participants moved because of the disaster. Loss of close relatives or friends was defined by asking the question “Did you lose a close relative or friend in the earthquake?” All damage variables were binary.

For community social capital, we used three subscales that had been validated in previous studies (Saito et al., 2017). These subscales examined three aspects of social capital, separately. The first subscale measured social cohesion, the second social participation, and the third reciprocity. Social cohesion was calculated as the mean of three 1- to 5-point Likert scales to the following questions (1 = “not at all,” 2 = “slightly,” 3 =“neutral,” 4 = “moderately,” and 5 =“very”): (1) “Do you think that people living in your community can be trusted in general?” (2) “Do you think people living in your community try to help others in most situations?,” and (3) “How attached are you to the community in which you live?” To assess social participation, participants were asked the following three questions: (1) “How often do you attend sports club activities?” (2) “How often do you attend hobby groups?,” and (3) “How often do you attend volunteer groups?” Social participation was the mean score of values from 1 to 6 to represent “not at all” to “every day.” Reciprocity was measured using the following yes/no questions: (1) “Do you listen to someone’s concerns and complaints?” (2) “Do you have someone who listens to your concerns and complaints?,” and (3) “Do you have someone who looks after you when you are sick for a few days?” Reciprocity mean scores ranged from 0 to 1, representing the expectation of items to which participants answered “yes.”

#### Covariates and mediator

With reference to previous studies, we controlled for sociodemographic variables including sex, marital status, education, equivalized income, and employment status (Deandrea et al., 2010; Dierking et al., 2016; Friedman et al., 2002; West et al., 2004). Baseline age, but not the time-varying age, was included to avoid collinearity with the survey time variable. Multimorbidity was controlled for by an index of 10 health conditions such as heart disease, hypertension, musculoskeletal disease, traumatic injury, eye disease, and so forth (Scheffer et al., 2008; Thiamwong et al., 2017). Analyses were also adjusted for smoking status and drinking status (Canada et al., 2020; Suzuki et al., 2002), as well as the dummy variable for school code to control for the physical environment (Lee et al., 2018).

We additionally introduced instrumental activities of daily living (IADLs), a measure of functional disability, into analyses as a potential mediator of the associations between disaster damage and fear of falling/falls. IADL was calculated as the mean score of answers of 0 (no) or 1 (yes) to 13 questions (e.g., “Can you go out alone by train or bus?”; Fujiwara et al., 2003). The diagram of survey time for all study variables is shown in Supplementary Figure 1.

### Statistical Analyses

We imputed the missing values of community social capital and covariates using the Markov chain Monte Carlo methods with mi command in Stata. We created 10 imputed data sets and used the mi estimate command to estimate model parameters from multiple imputed data.

Logistic regression models with random effects were used to analyze the panel data. All dependent variables were lagged behind exposures and covariates to establish temporality. For the main analyses, Model 1 only included disaster damage variables with covariates, Model 2 only included community social capital variables with covariates, and Model 3 included disaster damage, community social capital variables, and covariates. Sex-stratified analyses examined the heterogeneity between male and female participants. The Baron and Kenny method was used to determine the mediating effect. According to this method, the exposures (disaster damage) should have significant effects on the dependent variables (fear of falling and falls) and the mediating variable (IADL), separately. In the joint analyses of the mediating variable and exposures, the mediating variable should have significant effects on the dependent variables and the effects of exposures on the dependent variables should decrease (partial mediation) or disappear (full mediation; Baron et al., 1986).

In sensitivity analyses, we used nonimputed data and data from those participating in all four waves to confirm our results, separately. All analyses were performed using Stata (version 17). Statistical significance was set at *p* < .05 and all tests were two-tailed.

## Results

Tables 1 and 2 present the descriptive characteristics of the sample at baseline and disaster damage experienced by the Iwanuma cohort participants. Of the 4,957 valid responses in 2010, 56.4% were female. About a quarter were older than 80 and 65% were married. Individuals who had an equivalized income below 2 million JPY accounted for 40.0% of the sample. As for individuals’ health, the mean (standard deviation [*SD*]) of multimorbidity index was 1.6 (1.1). The mean (*SD*) of social cohesion, social participation, and reciprocity were 3.7 (0.7), 5.2 (1.1), and 0.9 (0.2). With respect to disaster damage for the 3,567 individuals in 2013, 57.3% reported home destruction and 5.9% experienced relocation. There were 23.2%, 12.8%, and 37.3% of the participants reported financial hardship, health care disruption, and loss of close relatives or friends, respectively.

**Table 1.** Baseline (2010) Characteristics Among the Iwanuma Cohort (*N* = 4,957)

Characteristics	Total (*N* = 4,957)		Female (*n* = 2,798)		Male (*n* = 2,159)	
	Mean (*SD*)	*N* (%)	Mean (*SD*)	*N* (%)	Mean (*SD*)	*N* (%)
Sociodemographics						
Age	74.8 (7.1)		75.5 (7.3)		73.9 (6.6)	
Marital status						
Married		3,220 (65.0)		1,407 (50.3)		1,813 (84.0)
Unmarried		1,524 (30.7)		1,247 (44.6)		277 (12.8)
Missing		213 (4.3)		144 (5.1)		69 (3.2)
Education						
9 years or less		1,811 (36.5)		1,065 (38.1)		746 (34.6)
10–12 years		1,963 (39.6)		1,155 (41.3)		808 (37.4)
13 years or more		927 (18.7)		397 (14.2)		530 (24.5)
Missing		256 (5.2)		181 (6.4)		75 (3.5)
Equivalised income						
<2 million JPY		1,982 (40.0)		1,083 (38.7)		899 (41.6)
2–4 million JPY		1,573 (31.7)		785 (28.1)		788 (36.5)
>4 million JPY		395 (8.0)		224 (8.0)		171 (7.9)
Missing		1,007 (20.3)		706 (25.2)		301 (14.0)
Employment status						
Have a paid job		700 (14.1)		250 (8.9)		450 (20.8)
Retired		2,742 (55.3)		1,315 (47.0)		1,427 (66.1)
Never had a job		850 (17.1)		716 (25.6)		134 (6.2)
Missing		665 (13.5)		517 (18.5)		148 (6.9)
School code ^a^						
Iwanuma-minami		1,293 (26.1)		730 (26.1)		563 (26.1)
Iwanuma-nishi		1,286 (25.9)		675 (24.1)		611 (28.3)
Iwanuma		1,492 (30.1)		864 (30.9)		628 (29.1)
Tamaura		886 (17.9)		529 (18.9)		357 (16.5)
Community social capital						
Social cohesion^b^	3.7 (0.7)		3.7 (0.7)		3.8 (0.7)	
Social participation^c^	5.2 (1.1)		5.2 (1.1)		5.2 (1.0)	
Reciprocity^d^	0.9 (0.2)		0.9 (0.2)		0.9 (0.2)	
Health and lifestyle						
Multimorbidity^e^	1.6 (1.1)		1.6 (1.1)		1.6 (1.1)	
Smoking status						
Current		504 (10.2)		83 (3.0)		421 (19.5)
Used to		1,255 (25.3)		116 (4.1)		1,139 (52.8)
Never		2,767 (55.8)		2,241 (80.1)		526 (24.4)
Missing		431 (8.7)		358 (12.8)		73 (3.3)
Drinking status						
Current		1,641 (33.1)		349 (12.5)		1,292 (59.8)
Used to		201 (4.1)		20 (0.7)		181 (8.4)
Never		2,991 (60.3)		2,333 (83.4)		658 (30.5)
Missing		124 (2.5)		96 (3.4)		28 (1.3)

*Notes*: JPY = Japanese Yen; *SD* = standard deviation..

^a^School code refers to the four elementary school districts in Iwanuma and the districts construct geographical environment where participants live.

^b^Social cohesion is the mean score of answers ranged from 1 (low) to 5 (high) to three questions.

^c^Social participation is the mean score of answers ranged from 1 (never) to 6 (everyday) to three questions.

^d^Reciprocity is the mean score of answers of 0 (no) or 1 (yes) to three questions.

^e^Multimorbidity is the aggregated score of answers of 0 (no) or 1 (yes) to 10 major geriatric diseases (cancer, heart disease, stroke, high blood pressure, diabetes, respiratory disease, musculoskeletal disease, eye disease, ear disease and traumatic injury).

**Table 2.** Disaster Damage Among the Iwanuma Cohort in 2013 (*N* = 3,567)

Disaster damage	Total (*n* = 3,567)	Female (*n* = 2,015)	Male (*n* = 1,552)
	*N* (%)	*N* (%)	*N* (%)
Home destruction			
Yes	2,043 (57.3)	1,168 (58.0)	875 (56.4)
No	1,423 (39.9)	779 (38.7)	644 (41.5)
Missing	101 (2.8)	68 (3.3)	33 (2.1)
Financial hardship			
Yes	829 (23.2)	463 (23.0)	366 (23.6)
No	2,652 (74.3)	1,494 (74.1)	1,158 (74.6)
Missing	86 (2.5)	58 (2.9)	28 (1.8)
Health care disruption			
Yes	457 (12.8)	294 (14.6)	163 (10.5)
No	3,110 (87.2)	1,721 (85.4)	1,389 (89.5)
Missing	0 (0.0)	0 (0.0)	0 (0.0)
Home relocation			
Yes	210 (5.9)	131 (6.5)	79 (5.1)
No	3,250 (91.1)	1,808 (89.7)	1,442 (92.9)
Missing	107 (3.0)	76 (3.8)	31 (2.0)
Loss of close relatives/friends			
Yes	1,329 (37.3)	779 (38.7)	550 (35.4)
No	2,238 (62.7)	1,236 (61.3)	1,002 (64.6)
Missing	0 (0.0)	0 (0.0)	0 (0.0)

*Notes*: Disaster damage was inquired in the follow-up survey in 2013 and included 3,567 valid respondents. Among them, there were 2,015 female respondents and 1,552 male respondents.

Table 3 reports results from logistic regression models evaluating the associations between disaster damage and community social capital with fear of falling and falls. In Model 3, financial hardship significantly predicted both fear of falling (odds ratio (OR), 1.75; 95% confidence interval (CI) [1.33, 2.28]) and falls (OR, 1.29; 95% CI [1.05, 1.58]), after adjusting for social capital, sociodemographic, health, and lifestyle variables. Health care disruption was significantly associated with fear of falling (OR, 1.54; 95% CI [1.11, 2.15]) but not falls (OR, 1.27; 95% CI [0.99, 1.62]) after full adjustment. Home destruction and loss of close relatives or friends were not statistically significant in the model. Home relocation was inversely associated with fear of falling (OR, 0.57; 95% CI [0.34, 0.94]). In terms of community social capital, after controlling for disaster damage variables and covariates, social cohesion was negatively associated with fear of falling (OR, 0.82; 95% CI [0.71, 0.95]) and falls (OR, 0.88; 95% CI [0.78, 0.98]). Social participation was positively associated with fear of falling (OR, 1.17; 95% CI [1.07, 1.28]) and falls (OR, 1.12; 95% CI [1.05, 1.20]). The estimates for reciprocity were not statistically significant. Overall, the results confirmed our hypothesis for the primary study aim that disaster damage predicted a higher risk of fear of falling and falls whereas the specific associations were different in terms of damage types. The hypothesis for the secondary aim was that each form of social capital would be associated with lower odds of fear of falling and falls. However, only social cohesion was protectively associated with fear of falling and falls.

**Table 3.** Logistic Regression Models Estimating Fear of Falling/Falls

Variable	Fear of falling						Falls					
	Model 1		Model 2		Model 3		Model 1		Model 2		Model 3	
	OR	95% CI	OR	95% CI	OR	95% CI	OR	95% CI	OR	95% CI	OR	95% CI
Disaster damage												
Home destruction (vs. no)	1.18	[0.95, 1.46]			1.22+	[0.98, 1.51]	1.13	[0.95, 1.34]			1.16^+^	[0.98, 1.38]
Financial hardship (vs. no)	1.81***	**[1.38, 2.37]**			1.75***	**[1.33, 2.28]**	1.32**	**[1.08, 1.63]**			1.29*	**[1.05, 1.58]**
Health care disruption (vs. no)	1.57**	**[1.13, 2.18]**			1.54*	**[1.11, 2.15]**	1.28*	**[1.002, 1.64]**			1.27^+^	[0.99, 1.62]
Home relocation (vs. no)	0.59*	**[0.36, 0.98]**			0.57*	**[0.34, 0.94]**	0.74	[0.50, 1.09]			0.71^+^	[0.48, 1.05]
Loss of close relatives/friends (vs. no)	0.97	[0.79, 1.20]			1.002	[0.81, 1.24]	1.05	[0.89, 1.24]			1.07	[0.91, 1.27]
Community social capital												
Social cohesion			0.82**	**[0.72, 0.94]**	0.82**	**[0.71, 0.95]**			0.89*	**[0.80, 0.99]**	0.88*	**[0.78, 0.98]**
Social participation			1.17***	**[1.08, 1.27]**	1.17***	**[1.07, 1.28]**			1.10**	**[1.03, 1.18]**	1.12**	**[1.05, 1.20]**
Reciprocity			1.11	[0.68, 1.80]	1.05	[0.62, 1.78]			0.76	[0.51, 1.12]	0.76	[0.51, 1.15]

*Notes*: CI = Confidence interval; OR = odds ratio. Model 1 included disaster damage variables and covariates. Model 2 included community social capital variables and covariates. Model 3 included disaster damage variables, community social capital variables, and covariates. All models controlled for covariates including multimorbidity, sex, marital status, education, equivalised income, employment status, baseline age, smoking status, drinking status, school code, and survey time. Bold items showed significant associations.

^+^ *p* < .10. *p < .05. **p < .01. ***p < .001.

**Figure 1.**
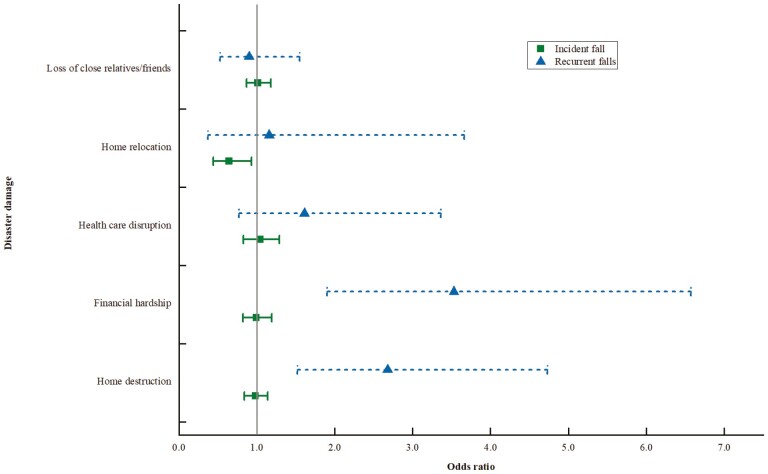
Odds ratios and 95% confidence intervals for the associations between disaster damage and incident fall and recurrent falls.

When we looked further into the two subtypes of falls, we found that disaster damage was more likely to be associated with recurrent falls (Figure 1). Home destruction (OR, 2.68; 95% CI [1.52, 4.73]) and financial hardship (OR, 3.53; 95% CI [1.90, 6.57]) were both significantly associated with recurrent falls after adjustment. There was no significant association between disaster damage with incident fall, except for home relocation (OR, 0.64; 95% CI [0.44, 0.93]).

**Figure 2.**
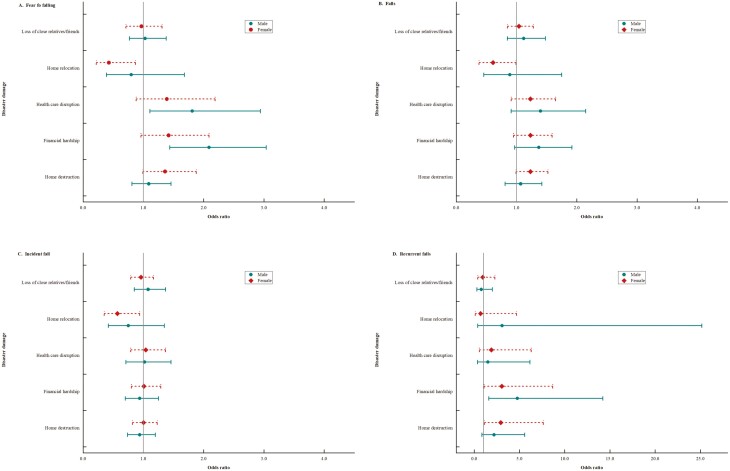
Sex-stratified odds ratios and 95% confidence intervals for the associations between disaster damage and fear of falling/falls/incident fall/recurrent falls.

Figure 2 shows the results of sex-stratified analyses of the associations between disaster exposures with four types of fall-related variables. For males, financial hardship was positively associated with fear of falling (OR, 2.09; 95% CI [1.44, 3.04]) and recurrent falls (OR, 4.76; 95% CI [1.60,14.21]), and health care disruption was associated with fear of falling (OR, 1.81; 95% CI [1.11, 2.94]). For females, the significant associations between home relocation with most fall-related variables were inverse (OR, 0.43, 95% CI [0.22, 0.87] for fear of falling; OR, 0.61, 95% CI [0.38, 0.99] for falls; OR, 0.57, 95% CI [0.35, 0.94] for incident fall); whereas home destruction (OR, 2.92, 95% CI [1.12, 7.63]) and financial hardship (OR, 3.05, 95% CI [1.07, 8.69]) increased the odds of recurrent falls.

For mediation analyses in Table 4, Model 2 shows that IADL was associated with financial hardship (ß, −0.02, 95% CI [−0.04, −0.01]) and health care disruption (ß, −0.02, 95% CI [−0.04, −0.01]), which significantly predicted the increased risk of fear of falling. In the joint regression of the mediating variable and the exposures in Model 3, IADL was shown as a significant predictor for fear of falling (OR, 0.06, 95% CI [0.03, 0.10]) and the predicting power of financial hardship (OR, 1.66, 95% CI [1.27, 2.16]) and health care disruption (OR, 1.43, 95% CI [1.03, 2.00]) was attenuated. Similar mediating effects of IADL occurred between the relationships of financial hardship and falls.

**Table 4.** Mediation Analyses for Fear of Falling/Falls Using IADL as the Mediator

Variables	Fear of falling						Falls					
	Model 1 (fear of falling)		Model 2 (IADL)		Model 3 (fear of falling)		Model 1 (falls)		Model 2 (IADL)		Model 3 (falls)	
	OR	95% CI	Beta	95% CI	OR	95% CI	OR	95% CI	Beta	95% CI	OR	95% CI
Disaster damage												
Home destruction (vs. no)	1.22+	[0.98, 1.51]	−0.003	[-0.01, 0.01]	1.20	[0.97, 1.49]	1.16+	[0.98, 1.38]	-0.003	[-0.01, 0.01]	1.15	[0.96, 1.36]
Financial hardship (vs. no)	1.75***	**[1.33, 2.28]**	−0.02***	**[−0.04, −0.01]**	1.66***	**[1.27, 2.16]**	1.29*	**[1.05, 1.58]**	−0.02***	**[−0.04, −0.01]**	1.25*	**[1.02, 1.54]**
Health care disruption (vs. no)	1.54*	**[1.11, 2.15]**	−0.02**	**[−0.04, −0.01]**	1.43*	**[1.03, 2.00]**	1.27+	[0.99, 1.62]	−0.02**	**[−0.04, −0.01]**	1.23	[0.96, 1.57]
Home relocation (vs. no)	0.57*	**[0.34, 0.94]**	−0.01	[−0.04, 0.01]	0.53*	**[0.32, 0.87]**	0.71+	[0.48, 1.05]	−0.01	[−0.04, 0.01]	0.69+	[0.47, 1.02]
Loss of close relatives/friends (vs. no)	1.002	[0.81, 1.24]	0.01*	**[0.003, 0.03]**	1.06	[0.86, 1.31]	1.07	[0.91, 1.27]	0.01*	**[0.003, 0.03]**	1.11	[0.94, 1.31]
Community social capital												
Social cohesion	0.82**	**[0.71, 0.95]**	0.01***	**[0.005, 0.02]**	0.86*	**[0.74, 0.99]**	0.88*	**[0.78, 0.98]**	0.01***	**[0.005, 0.02]**	0.90+	[0.81, 1.01]
Social participation	1.17***	**[1.07, 1.28]**	−0.02***	**[−0.022, −0.01]**	1.08+	[0.99, 1.19]	1.12**	**[1.05, 1.20]**	−0.02***	**[−0.02, −0.01]**	1.07+	[0.996, 1.15]
Reciprocity	1.05	[0.62, 1.78]	0.03**	**[0.01, 0.05]**	1.41	[0.83, 2.40]	0.76	[0.51, 1.15]	0.03**	**[0.01, 0.05]**	0.91	[0.60, 1.37]
Mediator												
IADL					0.06***	**[0.03, 0.10]**					0.22***	**[0.15, 0.32]**

*Notes*: CI = confidence interval; IADL = instrumental activity of daily living; OR = odds ratio. To test the mediating effect of IADL, the Baron and Kenny method (1986) was used. Model 1 examined the associations between disaster damage with fear of falling/falls. Model 2 examined the associations between disaster damage with IADL. Model 3 jointly regressed disaster damage and IADL on fear of falling/falls, and checked whether IADL was a significant predictor for fear of falling/falls and whether disaster damage’s predicting power was attenuated. All models controlled for multimorbidity, sex, marital status, education, equivalised income, employment status, baseline age, smoking status, drinking status, school code, and survey time. Bold items showed significant associations.

^+^ *p* < .10. *p < .05. **p < .01. ***p < .001.

For sensitivity analyses, results from data without multiple imputations and from those who participated in all follow-up studies are presented in Supplementary Tables 1 and 2, respectively. The primary results remained in the same direction, and significance changed a little, indicating that the substantive conclusions were unchanged.

## Discussion and Implications

To our knowledge, this is the first study to empirically demonstrate the long-term impact of disaster damage on fear of falling and falls. Using longitudinal data from a unique natural experiment, we found that financial hardship predicted higher odds of fear of falling and falls. Specifically, recurrent falls, rather than incident fall, were more strongly associated with home destruction and financial hardship. Unexpectedly, survivors appeared to benefit from relocation in terms of fall-related risks. IADL partly mediated the relationships linking disaster damage to fall problems of older people. Besides, community social cohesion was inversely linked to fear of falling and falls whereas social participation increased the risk of these issues.

Overall, our findings that disaster damage were persistently associated with fear of falling/falls aligned with the theoretical assumptions by the person–environment fit theory. When analyzing the health threats caused by the disaster, we differentiated among types of disaster damage. Disasters are associated with three broad categories of health threats besides injuries: (a) psychic trauma such as traumatic bereavement; (b) material loss such as property damage; and (c) ongoing life changes such as relocation (Hikichi et al., 2021; Norris et al., 2009). Consistent with the findings from previous research about disaster damage and individuals’ health (Li et al., 2018), we found that material loss (e.g., financial hardship) emerged as salient predictors of sustained fear of falling and falls during even 9 years after the disaster, whereas there was no evidence linking psychic trauma (e.g., from bereavement) with fear of falling or falls. The effects of material damage could be explained in two ways. First, damage to the physical structure of residences might increase the risk of falls. Second, loss of wealth probably contributed to the inability to afford resources supporting the maintenance of physical function. Our mediation analyses suggested that the associations between material damage and fear of falling/falls were partly mediated by IADL (Table 4), providing support for this interpretation. In this regard, previous studies showed associations between disaster damage and functional disability (Hikichi et al., 2021; Yamamoto et al., 2022). Our work advanced this line of research by further linking disaster damage to falls and pointing to the mediating effect of functional disability in this relationship. By contrast, bereavement might be a more familiar experience as people aged (Shimai, 2004). Thus, older adults might become more resilient about the loss of loved ones and recover over time.

In addition, our results showed that material loss from natural disasters might result in cumulative disadvantage in the long term. Recurrent falls, but not incident fall, were associated with disaster damage, suggesting a health trajectory shaped through disaster-related “insults” (Chen et al., 2010; Hayward et al., 2004). Early fall experiences might be traumatic, leading to a self-reinforcing pattern of physical decline (Maclean, 2010; Tomita et al., 2018). People with lower capacity were much more sensitive to the demands of the environment than people with higher competence (Gitlin et al., 2017; Lawton et al., 1968). Besides, given that incident fall is usually caused by some accident (Mak et al., 2010) while recurrent falls are more linked to persistent factors (Boye et al., 2015), material deterioration might cause some irreversible changes and increase survivors’ risk of recurrent falls.

Most prior studies treated residential relocation as a form of ongoing adversity (Norris et al., 2009), whereas our results showed that survivors, especially women, might benefit from relocation in terms of fall risks. Some research suggested that relocation might be a proactive strategy to enhance congruence between older adults’ abilities and their living environment (Golant, 2011; Granbom et al., 2019), which may provide a theoretical basis for our findings. Specifically, there were at least three possible explanations for the observation. First, relocated survivors, who left their original social network, might be unfamiliar with other residents in the new areas (Kawachi et al., 2020). In this case, people might socialize less outside, rendering fewer bumps and reducing risk of falls. Second, survivors could be at increased risk of falls due to abnormal gaits caused by posttraumatic worries (Hadjistavropoulos et al., 2007). Moving away from the original residence could provide placebo effects and help them get over their trauma. The third reason might stem from the changes in the physical environment. Of the 210 who relocated after the disaster, 17.6% moved to public prefabricated temporary housing villages (*kasetsu jyutaku* in Japanese), made up of single-story trailers, and hence reduced the risk of falling down the stairs.

Sex-stratified analyses showed that women were more influenced by home destruction and relocation, whereas men were more sensitive to financial hardship. The findings added to sex-stratified results from some large national surveys, showing that the associations between potential risk factors with falls differed between genders (Gale et al., 2016). Because older women in Japan might spend more time at home (Uji et al., 2006), house status could exert a larger impact on them.

With regards to community social capital, the results revealed that the effects of social cohesion were protective whereas social participation might increase fall risks. According to the person–environment fit theory, a harmonious social environment could enhance individual’s resilience (Whitehall et al., 2021). However, social participation usually means venturing outside the home and facing more uncertainties, which easily increases the risk of falls. Those effects of social participation were fully mediated by IADL. Hence, targeted intervention strategies can be conducted to prevent fear of falling and falls, which is perhaps one of the most important implications of this study. For instance, enhancing social cohesion by building trust, increasing attachment, and encouraging altruistic behaviors among community members, as well as the emphasis on self-protection when socially participating, may be essential ways to reduce fear of falling and falls. Social cohesion was more strongly correlated with fear of falling than actual falls. This might be true because falls are the consequence of actual behaviors whereas fear of falling is a psychological construct that involves risk perception (Friedman et al., 2002; Suzuki et al., 2002; Zhang et al., 2013). Hence, social cohesion may psychologically increase survivors’ confidence and sense of being protected, and thus decrease the extent of fear of falling (Dierking et al., 2016).

Our findings also contributed to the broader literature on the relationships between stressors with fear of falling and falls. Some studies reported that major life events and sudden stress increased the risk of falls (Fink et al., 2014; Moller et al., 2009). However, these prior studies on stressors and falls were usually limited by potential endogeneity problems in that unobserved confounders could bias the results. Our study, a natural experiment with longitudinal data of up to 9 years of follow-up, was less likely to have endogeneity problems as the stressors were imposed by a natural disaster and were thus exogenous. Hence, the conclusion can deepen our understanding about which kinds of stressors are associated with fear of falling and falls and inform interventions to prevent older adults from fear of falling and falls.

This study has limitations. First, this study might have selection bias due to the 59% response rate at the baseline survey. Nonetheless, prior research has demonstrated that the Iwanuma cohort at baseline was demographically representative of the whole older adult population in Iwanuma City (Hikichi et al., 2021). Second, 749 participants died between the 2011 disaster and the 2020 survey. These people were likely to be those who were most severely affected by the disaster and might have a higher prevalence of fear of falling and/or falls. In this case, we are likely to be underestimating our interest associations. Third, fear of falling and falls were self-reported, and we had no more details about the intensity of falls (e.g., minor falls or life-threatening falls) and physical environment. Future research could consider using more objective measures such as hip-mounted triaxial accelerometer sensors, evaluating the intensity of falls, and investigating the physical living conditions.

In conclusion, material hardship in the aftermath of disasters was associated with fear of falling and falls in the long term. Moreover, recurrent falls were much more affected by material loss through a cumulative disadvantage process. Other life changes after the disaster were not always negative events because survivors, especially women, might benefit from relocation in terms of fall-related risks. Functional disability was one of the potential mediators linking disaster damage with fear of falling and falls. Besides, fear of falling, a kind of risk perception, was significantly buffered by social cohesion. The findings could inform targeted strategies to mitigate lingering fall-related problems among older disaster survivors. For instance, long-term recovery efforts for older adults could be directed to the provision of material support such as financial support, health care, and home repairment, as well as strengthening social attachment. Attention should also be paid to survivors who have fallen previously. These efforts would contribute to stronger resilience for disaster-affected population and communities.

## Supplementary Material

igad020_suppl_Supplementary_Material
