# Supplementary material for: Longitudinal Associations Between Disaster Damage and Falls/Fear of Falling in Older Adults: 9-Year Follow-Up of Survivors of the 2011 Great East Japan Earthquake and Tsunami
Source: Innov Aging. 2023 Mar 1;7(3):igad020. doi: 10.1093/geroni/igad020 (PMC10089294; doi:10.1093/geroni/igad020)
Supplement: igad020_suppl_Supplementary_Material [file igad020_suppl_supplementary_material.docx]

*Innovation in Aging* Online Supplementary Material: Yuhang Wang, Chenggang Zhang, Hiroyuki Hikichi, Ichiro Kawachi, & Xiaoyu Li.

Longitudinal associations between disaster damage and falls/fear of falling in older adults: 9-year follow-up of survivors of the 2011 Great East Japan Earthquake and Tsunami.

**Supplementary Table 1. Logistic regression models** **estimating fall-related outcomes with original non-imputed data**

| **Variables** | **Fear of falling** | | | | | | **Falls** | | | | | |
| --- | --- | --- | --- | --- | --- | --- | --- | --- | --- | --- | --- | --- |
|  | **Total** | | **Sex-stratified analyses** | | | | **Total** | | **Sex-stratified analyses** | | | |
|  |  |  | Male | | Female | |  |  | **Male** | | **Female** | |
|  | **OR** | **95% CI** | **OR** | **95% CI** | **OR** | **95% CI** | **OR** | **95% CI** | **OR** | **95% CI** | **OR** | **95% CI** |
| **Disaster damage** |  |  |  |  |  |  |  |  |  |  |  |  |
| Home destruction (vs. no) | 1.04 | [0.79,1.35] | 0.96 | [0.68,1.35] | 1.17 | [0.76,1.80] | 1.16 | [0.93,1.43] | 1.09 | [0.78,1.52] | 1.20 | [0.91,1.58] |
| Financial hardship (vs. no) | **1.91***** | **[1.36,2.68]** | **2.35***** | **[1.51,3.65]** | 1.35 | [0.78,2.32] | **1.38*** | **[1.06,1.80]** | **1.53*** | **[1.02,2.30]** | 1.25 | [0.88,1.76] |
| Health care disruption (vs. no) | **1.80**** | **[1.18,2.75]** | **1.85*** | **[1.05,3.27]** | 1.83+ | [0.96,3.49] | 1.24 | [0.91,1.70] | 1.36 | [0.82,2.27] | 1.19 | [0.81,1.76] |
| Home relocation (vs. no) | **0.40**** | **[0.20,0.80]** | 0.56 | [0.22,1.41] | **0.27*** | **[0.10,0.78]** | 0.64 | [0.38,1.10] | 0.94 | [0.40,2.22] | **0.49*** | **[0.25,0.97]** |
| Loss of close relatives/friends (vs. no) | 0.99 | [0.76,1.28] | 1.01 | [0.72,1.41] | 0.97 | [0.64,1.48] | 1.13 | [0.92,1.39] | 1.09 | [0.78,1.52] | 1.16 | [0.88,1.51] |
| **Community social capital** |  |  |  |  |  |  |  |  |  |  |  |  |
| Social cohesion | **0.77**** | **[0.64,0.93]** | 0.88 | [0.69,1.13] | **0.63**** | **[0.46,0.86]** | 0.87+ | [0.75,1.01] | 0.82 | [0.65,1.04] | 0.90 | [0.74,1.10] |
| Social participation | **1.21***** | **[1.08,1.35]** | **1.24**** | **[1.08,1.43]** | 1.16 | [0.97,1.38] | **1.13**** | **[1.03,1.24]** | **1.24**** | **[1.07,1.43]** | 1.06 | [0.94,1.20] |
| Reciprocity | 1.03 | [0.48,2.21] | 1.05 | [0.44,2.50] | 0.86 | [0.18,4.07] | 0.66 | [0.36,1.21] | 0.72 | [0.32,1.64] | 0.62 | [0.25,1.54] |

**Supplementary Table 1. Logistic regression models** **estimating fall-related outcomes with original non-imputed data (continued)**

| **Variables** | **Incident fall** | | | | | | **Recurrent falls** | | | | | |
| --- | --- | --- | --- | --- | --- | --- | --- | --- | --- | --- | --- | --- |
|  | **Total** | | **Sex-stratified analyses** | | | | **Total** | | **Sex-stratified analyses** | | | |
|  |  |  | **Male** | | **Female** | |  |  | **Male** | | **Female** | |
|  | **OR** | **95% CI** | **OR** | **95% CI** | **OR** | **95% CI** | **OR** | **95% CI** | **OR** | **95% CI** | **OR** | **95% CI** |
| **Disaster damage** |  |  |  |  |  |  |  |  |  |  |  |  |
| Home destruction (vs. no) | 1.05 | [0.86,1.27] | 0.97 | [0.73,1.28] | 1.12 | [0.86,1.46] | **2.85**** | **[1.42,5.75]** | 2.50+ | [0.86,7.27] | 2.27+ | [0.90,5.73] |
| Financial hardship (vs. no) | 0.95 | [0.75,1.21] | 0.86 | [0.61,1.22] | 1.04 | [0.74,1.44] | **5.79***** | **[2.65,12.68]** | **5.66**** | **[1.73,18.51]** | **3.55*** | **[1.23,10.19]** |
| Health care disruption (vs. no) | 1.07 | [0.81,1.40] | 1.11 | [0.73,1.70] | 1.01 | [0.70,1.47] | 1.23 | [0.48,3.19] | 1.14 | [0.27,4.94] | 1.39 | [0.42,4.60] |
| Home relocation (vs. no) | 0.73 | [0.44,1.24] | 0.89 | [0.42,1.89] | 0.61 | [0.29,1.27] | 1.26 | [0.26,6.04] | 5.28 | [0.55,50.42] | 0.46 | [0.06,3.71] |
| Loss of close relatives/friends (vs. no) | 1.12 | [0.93,1.35] | 1.11 | [0.84,1.46] | 1.10 | [0.85,1.42] | 0.84 | [0.42,1.65] | 0.71 | [0.26,1.99] | 1.02 | [0.42,2.46] |
| **Community social capital** |  |  |  |  |  |  |  |  |  |  |  |  |
| Social cohesion | 0.92 | [0.80,1.07] | 0.84 | [0.68,1.04] | 1.04 | [0.85,1.28] | 0.79 | [0.50,1.26] | 1.19 | [0.60,2.35] | 0.60+ | [0.33,1.08] |
| Social participation | 0.95 | [0.88,1.04] | 1.02 | [0.90,1.16] | 0.92 | [0.82,1.04] | **1.57**** | **[1.15,2.14]** | **1.80*** | **[1.10,2.94]** | 1.25 | [0.83,1.87] |
| Reciprocity | 1.08 | [0.58,2.03] | 0.88 | [0.40,1.91] | 1.87 | [0.63,5.57] | 0.65 | [0.11,3.68] | 1.10 | [0.13,9.58] | 0.34 | [0.03,3.59] |

*Note.* OR = Odds ratio, CI = Confidence interval. For comparison with the main analyses, we used the original data without imputation in these models. Namely, those observations with any missing data were not used in the analyses. All models controlled for multi-morbidity, sex, marital status, education, equivalised income, employment status, baseline age, smoking status, drinking status, school code, and survey time.

+p < .10, *p < .05, **p < .01, ***p < .001.

**Supplementary Table 2. Logistic regression models estimating fall-related outcomes (with imputation) using samples participating all four waves**

| **Variables** | **Fear of falling** | | | | | | **Falls** | | | | | |
| --- | --- | --- | --- | --- | --- | --- | --- | --- | --- | --- | --- | --- |
|  | **Total** | | **Sex-stratified analyses** | | | | **Total** | | **Sex-stratified analyses** | | | |
|  |  |  | **Male** | | **Female** | |  |  | **Male** | | **Female** | |
|  | **OR** | **95% CI** | **OR** | **95% CI** | **OR** | **95% CI** | **OR** | **95% CI** | **OR** | **95% CI** | **OR** | **95% CI** |
| **Disaster damage** |  |  |  |  |  |  |  |  |  |  |  |  |
| Home destruction (vs. no) | 1.12 | [0.86,1.46] | 1.01 | [0.70,1.44] | 1.24 | [0.84,1.84] | 1.10 | [0.89,1.37] | 1.004 | [0.71,1.43] | 1.17 | [0.89,1.53] |
| Financial hardship (vs. no) | **1.55**** | **[1.12,2.15]** | **1.78*** | **[1.12,2.83]** | 1.33 | [0.83,2.13] | 1.26+ | [0.98,1.63] | 1.30 | [0.84,2.01] | 1.23 | [0.90,1.69] |
| Health care disruption (vs. no) | 1.28 | [0.85,1.94] | 1.69 | [0.90,3.17] | 1.07 | [0.61,1.88] | 1.15 | [0.84,1.57] | 1.15 | [0.64,2.03] | 1.16 | [0.80,1.68] |
| Home relocation (vs. no) | 0.73 | [0.37,1.44] | 0.82 | [0.30,2.26] | 0.66 | [0.26,1.65] | 0.62+ | [0.37,1.05] | 0.62 | [0.23,1.65] | 0.59+ | [0.32,1.10] |
| Loss of close relatives/friends (vs. no) | 0.97 | [0.75,1.25] | 1.01 | [0.71,1.44] | 0.94 | [0.65,1.35] | **1.24*** | **[1.01,1.52]** | 1.23 | [0.88,1.74] | 1.25+ | [0.97,1.61] |
| **Community social capital** |  |  |  |  |  |  |  |  |  |  |  |  |
| Social cohesion | **0.81*** | **[0.68,0.96]** | 0.84 | [0.66,1.07] | **0.77*** | **[0.61,0.99]** | 0.87+ | [0.76,1.002] | 0.89 | [0.71,1.12] | 0.86+ | [0.72,1.02] |
| Social participation | **1.14**** | **[1.04,1.27]** | **1.22**** | **[1.06,1.41]** | 1.08 | [0.94,1.24] | 1.09+ | [0.999,1.18] | **1.17*** | **[1.01,1.34]** | 1.04 | [0.94,1.15] |
| Reciprocity | 1.43 | [0.73,2.79] | 1.60 | [0.72,3.54] | 0.97 | [0.28,3.35] | 1.05 | [0.61,1.79] | 1.05 | [0.49,2.26] | 1.10 | [0.50,2.39] |

**Supplementary Table 2. Logistic regression models estimating fall-related outcomes (with imputation) using samples participating all four waves (continued)**

| **Variables** | **Incident fall** | | | | | | **Recurrent falls** | | | | | |
| --- | --- | --- | --- | --- | --- | --- | --- | --- | --- | --- | --- | --- |
|  | **Total** | | **Sex-stratified analyses** | | | | **Total** | | **Sex-stratified analyses** | | | |
|  |  |  | **Male** | | **Female** | |  |  | **Male** | | **Female** | |
|  | **OR** | **95% CI** | **OR** | **95% CI** | **OR** | **95% CI** | **OR** | **95% CI** | **OR** | **95% CI** | **OR** | **95% CI** |
| **Disaster damage** |  |  |  |  |  |  |  |  |  |  |  |  |
| Home destruction (vs. no) | 0.97 | [0.81,1.17] | 1.01 | [0.75,1.36] | 0.94 | [0.74,1.20] | **2.78**** | **[1.30,5.93]** | 2.17 | [0.71,6.64] | **4.13**** | **[1.42,12.04]** |
| Financial hardship (vs. no) | 0.89 | [0.71,1.12] | 0.88 | [0.61,1.26] | 0.91 | [0.68,1.22] | **5.92***** | **[2.65,13.19]** | **10.66***** | **[2.98,38.13]** | **6.49**** | **[2.13,19.80]** |
| Health care disruption (vs. no) | 1.03 | [0.78,1.35] | 1.02 | [0.64,1.65] | 1.03 | [0.73,1.44] | 1.33 | [0.49,3.61] | 0.56 | [0.08,3.68] | 2.06 | [0.55,7.67] |
| Home relocation (vs. no) | 0.73 | [0.45,1.17] | 0.61 | [0.26,1.41] | 0.8 | [0.44,1.43] | 0.85 | [0.17,4.27] | 3.34 | [0.23,47.98] | 0.53 | [0.06,4.57] |
| Loss of close relatives/friends (vs. no) | 1.02 | [0.86,1.22] | 1.07 | [0.81,1.42] | 0.99 | [0.79,1.24] | 1.27 | [0.64,2.52] | 1.14 | [0.39,3.34] | 1.28 | [0.50,3.26] |
| **Community social capital** |  |  |  |  |  |  |  |  |  |  |  |  |
| Social cohesion | **0.81**** | **[0.71,0.92]** | **0.78*** | **[0.64,0.97]** | **0.83*** | **[0.69,0.98]** | 0.999 | [0.65,1.53] | 2.08+ | [0.95,4.54] | 0.69 | [0.40,1.20] |
| Social participation | 0.97 | [0.90,1.05] | 1.06 | [0.92,1.21] | 0.93 | [0.84,1.03] | 1.18 | [0.90,1.55] | 1.59+ | [0.97,2.63] | 1.01 | [0.71,1.43] |
| Reciprocity | 1.34 | [0.73,2.45] | 0.75 | [0.35,1.58] | **3.83*** | **[1.29,11.39]** | 2.68 | [0.53,13.70] | 3.23 | [0.33,31.63] | 4.24 | [0.31,58.60] |

*Note.* OR = Odds ratio, CI = Confidence interval. For comparison with main analyses, we identified samples participating all four waves' researches (N = 2,047) and then imputed the social capital variables and covariates as stated. All models controlled for multi-morbidity, sex, marital status, education, equivalised income, employment status, baseline age, smoking status, drinking status, school code, and survey time.

+p < .10, *p < .05, **p < .01, ***p < .001.

**Supplementary Figure 1. Diagram of survey time for all study variables**
